# Multi-parametric MRI zone-specific diagnostic model performance compared with experienced radiologists for detection of prostate cancer

**DOI:** 10.1007/s00330-018-5799-y

**Published:** 2018-11-19

**Authors:** Nikolaos Dikaios, Francesco Giganti, Harbir S. Sidhu, Edward W. Johnston, Mrishta B. Appayya, Lucy Simmons, Alex Freeman, Hashim U. Ahmed, David Atkinson, Shonit Punwani

**Affiliations:** 1 Centre for Medical Imaging, University College London, 2nd floor, Charles Bell House, 43-45 Foley Street, London, W1W 7TS UK; 2 Centre for Vision, Speech and Signal Processing, University of Surrey, 388 Stag Hill, Guildford, GU2 7XH UK; 3 Departments of Radiology, University College London Hospital, 235 Euston Road, London, NW1 2BU UK; 4 Division of Surgery & Interventional Science, University College London, London, UK; 5 Research Department of Urology, Division of Surgery and Interventional Science, University College London, London, NW1 2PG UK; 6 Department of Histopathology, University College London Hospital, London, NW1 2PG UK; 7 Department of Surgery and Cancer, Imperial College London, London, UK

**Keywords:** Magnetic resonance imaging, Prostatic neoplasms, Diagnosis, Computer-assisted, Logistic models

## Abstract

**Objectives:**

Compare the performance of zone-specific multi-parametric-MRI (mp-MRI) diagnostic models in prostate cancer detection with experienced radiologists.

**Methods:**

A single-centre, IRB approved, prospective STARD compliant 3 T MRI test dataset of 203 patients was generated to test validity and generalisability of previously reported 1.5 T mp-MRI diagnostic models. All patients included within the test dataset underwent 3 T mp-MRI, comprising T2, diffusion-weighted and dynamic contrast-enhanced imaging followed by transperineal template ± targeted index lesion biopsy. Separate diagnostic models (transition zone (TZ) and peripheral zone (PZ)) were applied to respective zones. Sensitivity/specificity and the area under the receiver operating characteristic curve (ROC-AUC) were calculated for the two zone-specific models. Two radiologists (A and B) independently Likert scored test 3 T mp-MRI dataset, allowing ROC analysis for each radiologist for each prostate zone.

**Results:**

Diagnostic models applied to the test dataset demonstrated a ROC-AUC = 0.74 (95% CI 0.67–0.81) in the PZ and 0.68 (95% CI 0.61–0.75) in the TZ. Radiologist A/B had a ROC-AUC = 0.78/0.74 in the PZ and 0.69/0.69 in the TZ. Radiologists A and B each scored 51 patients in the PZ and 41 and 45 patients respectively in the TZ as Likert 3. The PZ model demonstrated a ROC-AUC = 0.65/0.67 for the patients Likert scored as indeterminate by radiologist A/B respectively, whereas the TZ model demonstrated a ROC-AUC = 0.74/0.69.

**Conclusion:**

Zone-specific mp-MRI diagnostic models demonstrate generalisability between 1.5 and 3 T mp-MRI protocols and show similar classification performance to experienced radiologists for prostate cancer detection. Results also indicate the ability of diagnostic models to classify cases with an indeterminate radiologist score.

**Key Points:**

*• MRI diagnostic models had similar performance to experienced radiologists for classification of prostate cancer.*

*• MRI diagnostic models may help radiologists classify tumour in patients with indeterminate Likert 3 scores.*

## Introduction

Multi-parametric MRI (mp-MRI) has heralded a paradigm shift in the management of prostate cancer. It is now commonly employed to localise suspicious areas within the prostate and facilitate targeted histological sampling [1]. Nevertheless, mp-MRI remains an imperfect test. For example, between 30 and 40% of mp-MRI studies, even by experienced radiologists, are scored as indeterminate for cancer (Likert/PIRADS 3) [2, 3]. An indeterminate mp-MRI confers little benefit. Most patients with indeterminate mp-MRI-scored studies do not have cancer within the gland [3]. However, a significant minority harbour small volume Gleason 3 + 4 disease or more widespread Gleason 3 + 3 disease [4]. An indeterminate study results in a management dilemma of whether to perform a biopsy. Not performing the biopsy risks underdiagnosing patients with significant prostate cancer, whilst performing a biopsy risks over the investigation of patients with likely insignificant or no cancer. Furthermore, mp-MRI also misses approximately 10% of cases of significant prostate tumour [4]. There remains a need to improve the performance of mp-MRI.

One potential approach to address these challenges has been to develop diagnostic models based on quantitative mp-MRI metrics. For example, we have previously derived zone-specific logistic regression (LR) models for classification of significant prostate cancer [5, 6]. Others have also developed similar models [7–9]. However, whilst studies based on internal validations suggest an overall good performance, it is recognised that this performance may be an overestimate and not generalisable to other (external) datasets.

Within this study, we aimed to assess the external validity of our previously derived and internally validated mp-MRI LR models [5, 6]. Specifically, we apply the zone-specific LR models, derived on a Siemens 1.5 T mp-MRI dataset, for the classification of an independent cohort of patients imaged using a Philips 3 T scanner.

In order to evaluate the potential clinical value of LR models, we compare their overall performance against experienced radiologists for classification of patients with significant prostate cancer and determine whether LR models could be applied for the classification of indeterminate (Likert 3/5) scored cases.

## Material and methods

Our local institutional review board approved the study and waived the requirement for individual consent for retrospective analysis of patient data collected as part of clinical trials/routine care (R&D No: 12/0195; date: 16 July 2012).

### Patient population

A single centre, IRB approved, prospective STARD compliant trial dataset of 330 patients [4] was reviewed. Three hundred thirty men (median age of 63 years, interquartile range, IQR [42–83]; median prostate-specific antigen (PSA) of 7.4 ng/ml, IQR [0.7–58.05]), with previous negative/ non-significant prostate disease on transrectal ultrasound (TRUS) biopsies, but in whom a clinical suspicion of prostate cancer remained, were consecutively enrolled from 11 January 2012 to 29 January 2014. All patients underwent 3 T mp-MRI (Achieva, Philips Healthcare) of the prostate. All studies were prospectively scored using a Likert scale by an experienced radiologist (radiologist A; with 14 years of experience in prostate mp-MRI and reporting more than 500 mp-MRI scans/year). Patients then underwent transperineal template mapping biopsies of the whole gland (irrespective or radiologist report) ± MR-targeted biopsy of a suspected index lesion (based on the radiologist report) as described within the PICTURE study protocol [4]. In summary, mapping using 5 mm sampling was obtained using core needles inserted via a brachytherapy grid fixed on a stepper. In most prostates, two biopsies at each grid point were required to sample the full craniocaudal gland length. Two to three targeted biopsies were performed for the mp-MRI index lesion-scored Likert 3 or above. In total, 90% of the patients underwent the additional targeted biopsy.

**Fig. 1:**
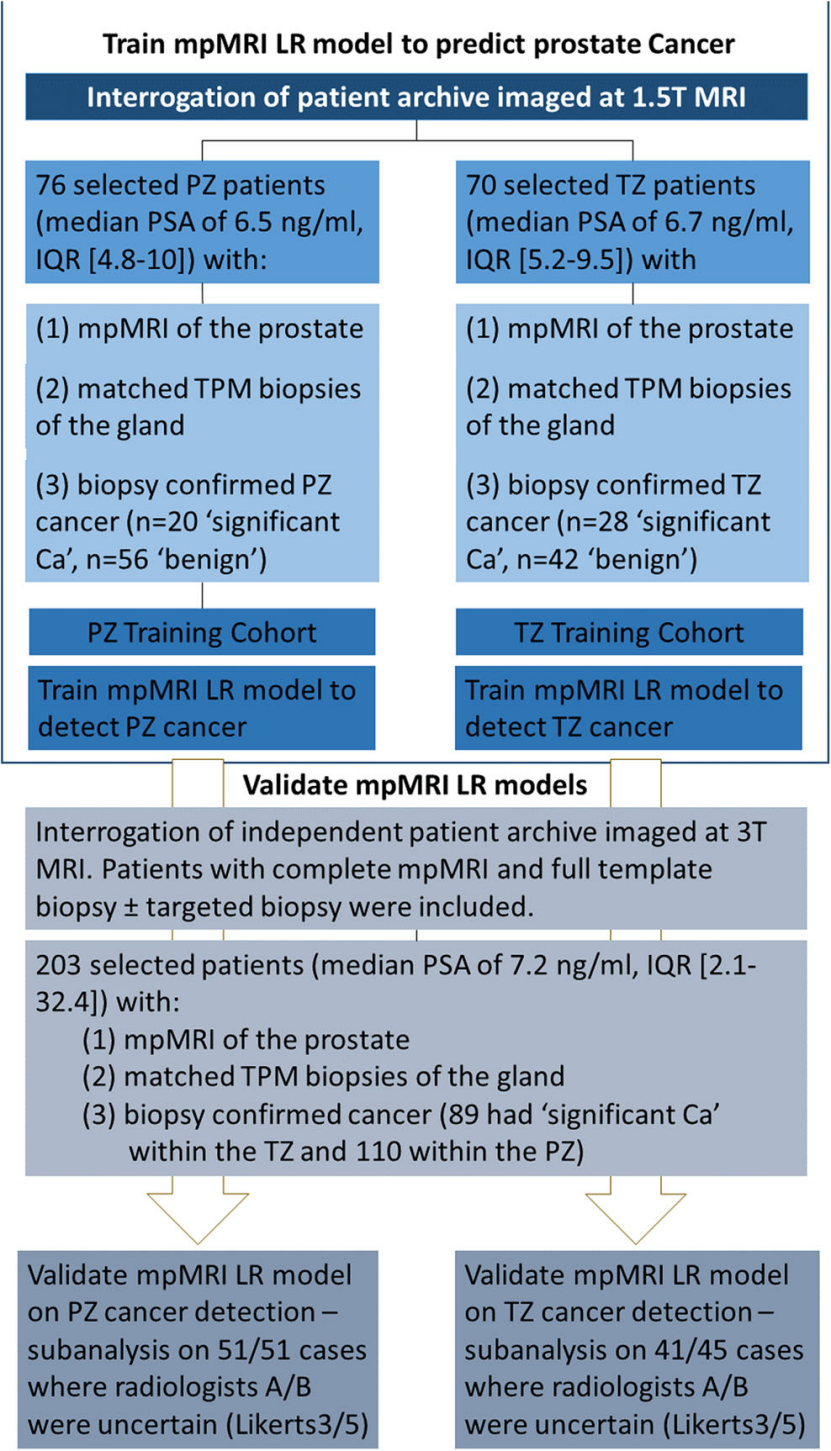
Flowchart outlining the study

To derive a complete dataset of patients scanned at 3 T, men were selected with the following criteria: (i) a full template biopsy ± targeted biopsy and (ii) a complete 3 T mp-MRI comprising of T2W, diffusion-weighted (DW) and dynamic contrast-enhanced (DCE) imaging. One hundred twenty-seven patients did not meet the inclusion criteria; 5 had mp-MRI performed at 1.5 T, 11 had incomplete mp-MRI datasets, 71 did not undergo the full template biopsy due to clinical circumstances, 13 patients were excluded due to post-biopsy artefacts, 12 patients were excluded due to distorted DWI and 15 patients were excluded where cancer was present in both the PZ and the TZ (Fig. 1). Two hundred three patients with median age 65.4 years (interquartile range, IQR = 42.9–86) and median PSA of 7.2 ng/ml (IQR = 2.1–32.4) formed the final test dataset.

Of the 203 men included in the final dataset, 89 had biopsy-confirmed significant cancer (see the “Histological reference standard” section) within the TZ and 110 biopsies confirmed significant cancer within the PZ. Median PSA of patients with significant cancer within the PZ and TZ was 7.22 ng/ml and 6.52 ng/ml respectively. Median PSA of patients with benign histology or non-significant cancer only within the PZ and TZ was 7.02 ng/ml and 7.77 ng/ml respectively.

A second radiologist (radiologist B, with 5 years of experience in prostate mp-MRI and reporting more than 1200 mp-MRI scans/year) retrospectively re-reported each of the 203 patient’s study, unaware of radiologist A’s original report and histological results.

As Likert scoring has been prospectively validated within multicentre PROMIS trial [10] and is the scoring method recommended by UK consensus [11], both radiologists (A and B) used the Likert scale to score the presence of significant disease within each prostate zone.

### Multi-parametric MRI protocol

The full details of the mp-MRI performed as part of the PICTURE trial have been previously reported [4]. In brief, MRI was performed on a single 3 T scanner (Achieva, Philips Healthcare) using a 32-channel cardiac phased-array coil. Prior to imaging, 0.2 mg/kg (up to 20 mg) of a spasmolytic agent (Buscopan; Boehringer Ingelheim) was administered intravenously to reduce bowel peristalsis. Axial and coronal T2-weighted images were acquired with TR/TE = 5407/100 ms, flip angle = 90°, field of view = 180 mm, a 3-mm slice thickness and slice centre-to-centre separation of 3 mm. Axial DW images were acquired at *b* = 0, 150, 500, 1000, and 2000 s/mm^2^ with TR/TE = 2753/80 ms, flip angle = 90°, field of view = 220 mm and a 5-mm slice thickness. A high *b* value at *b* = 2000 s/mm^2^ was included to evaluate the interstitial free water and permeability. An axial apparent diffusion coefficient (ADC) map was generated automatically from DW images at *b* = 0, 150, 500, and 1000 s/mm^2^. DCE was performed with a T1-weighted volumetric sequence (TR/TE = 5.8/28 ms, flip angle = 10°, field of view = 180 mm, slice thickness = 3 mm, temporal resolution of 15 s) before and after intravenous administration of at least 0.1 mmol/kg gadolinium meglumine contrast agent (Dotarem®, Guerbet) at a rate of 3 ml/s via power injector, followed by 20 ml saline bolus at the same rate.

### Radiologist Likert scoring

**Fig. 2:**
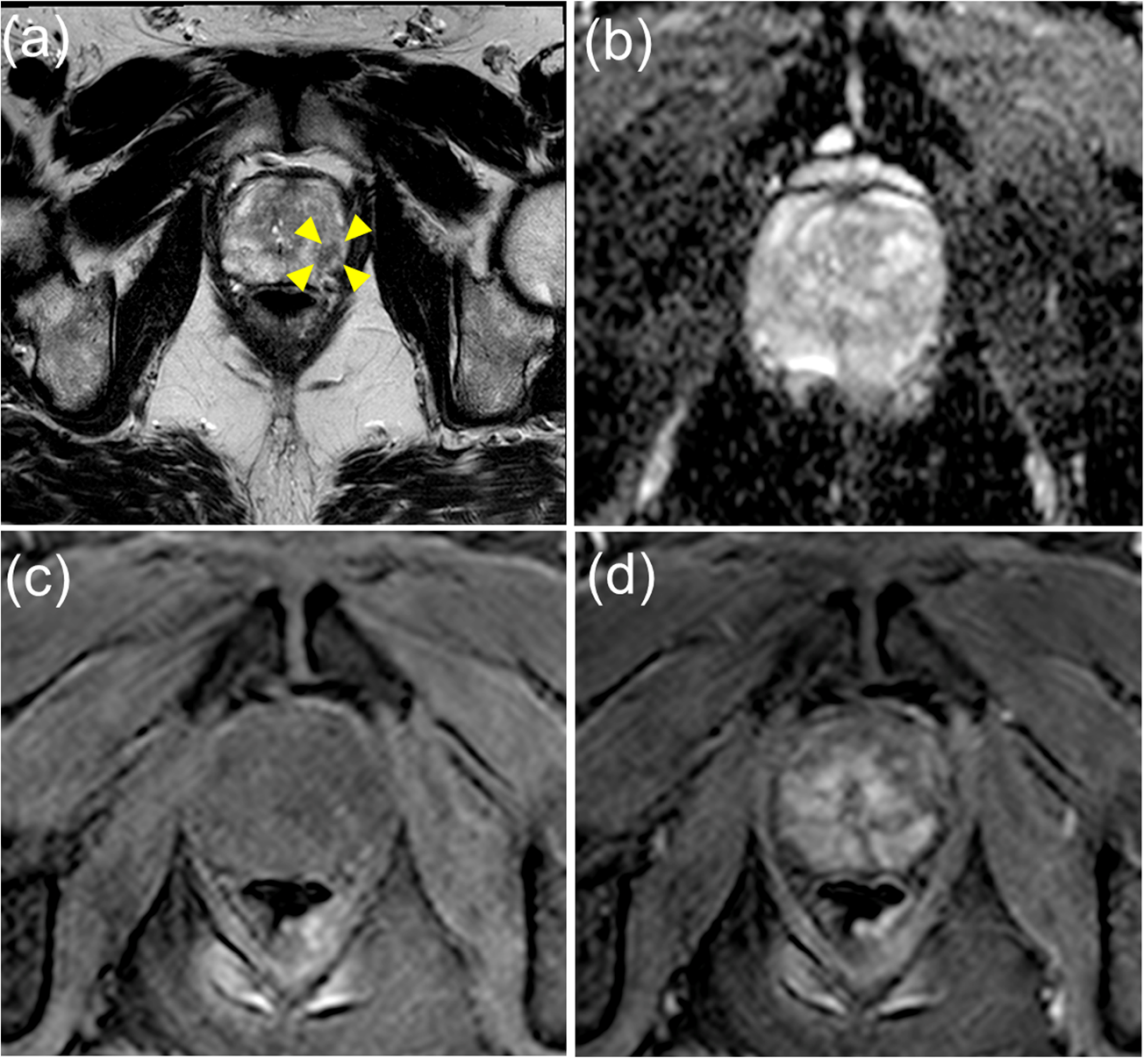
Axial multi-parametric MR images (**a** T2-weighted, **b** apparent diffusion coefficient map, **c** pre-contrast T1 and **d** early post-contrast T1) of a region in the peripheral zone scored as equivocal (diffused lesion; positive tumour TPM biopsy) by the radiologists

**Fig. 3:**
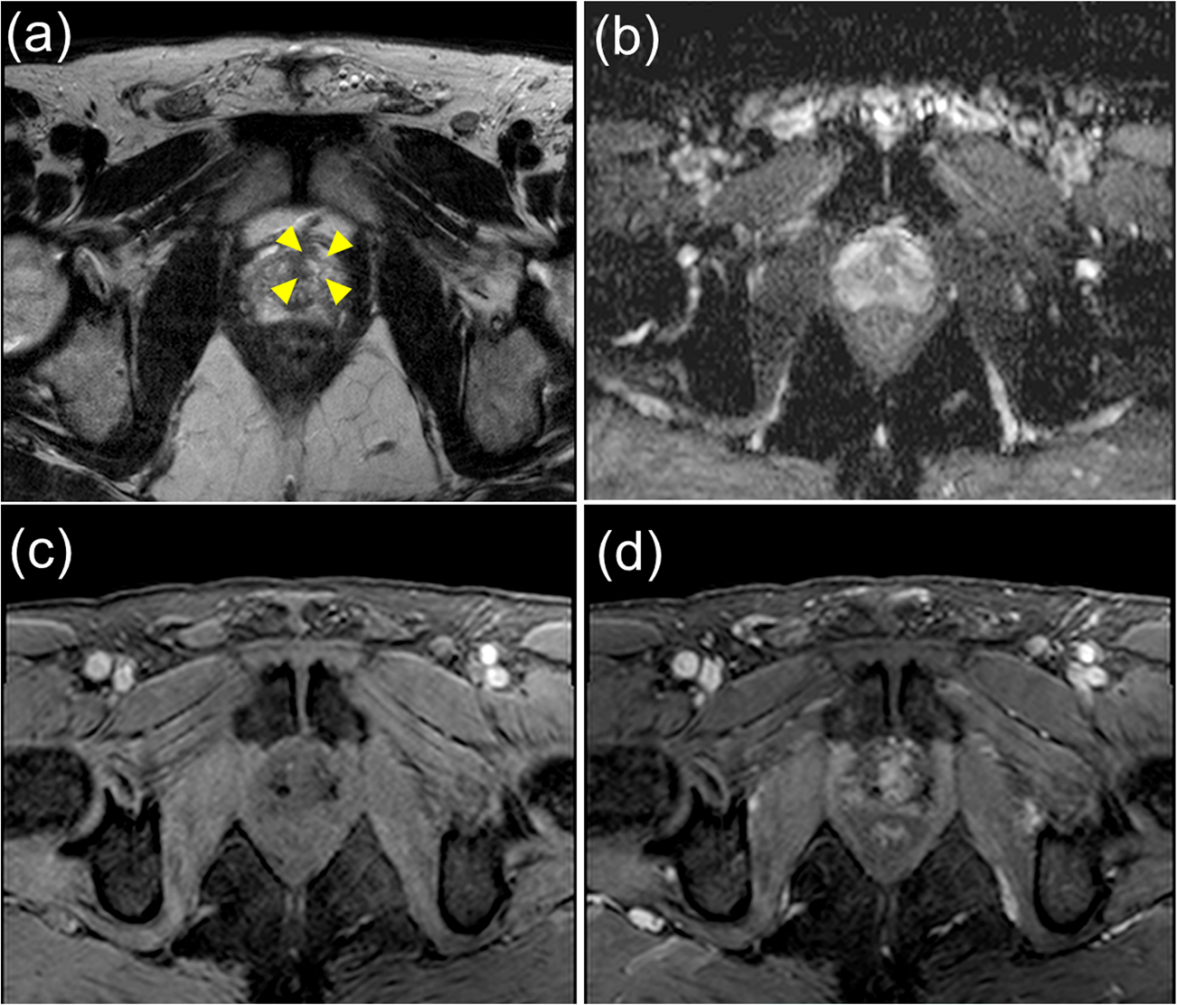
Axial multi-parametric MR images (**a** T2-weighted, **b** apparent diffusion coefficient map, **c** pre-contrast T1 and **d** early post-contrast T1) of a region (yellow arrows) in the transition zone scored as equivocal (positive tumour TPM biopsy) by the radiologists

Radiologists used a 12-segment (anterior/posterior, left/right, and division into the apical/middle/basal thirds) prostate pictorial reporting proforma to provide Likert scores for each segment together with drawing and scoring any identified focal lesions. For each patient, the highest Likert score given by each radiologist for each zone is summarised in Table 1. Figures 2 and 3 (in the PZ and the TZ respectively) show examples of the regions with positive template mapping biopsy (TPM) biopsy that were independently scored as indeterminate (Likert = 3) by both experienced radiologists.

**Table 1:** Radiologists A and B Likert scoring for the presence of significant cancer within the PZ and TZ in the cohort of patients scanned at 3 T

	Distribution of PZ Likert scores						
		Radiologist B Likert score					
		1	2	3	4	5	Total
Radiologist A Likert score	1	0	1	0	0	1	2
	2	0	31	9	0	5	45
	3	0	6	37	4	4	51
	4	0	3	3	31	12	49
	5	0	1	2	6	47	56
Total		0	42	51	41	69	203
	Distribution of TZ Likert Scores						
		Radiologist B Likert score					
		1	2	3	4	5	Total
Radiologist A Likert score	1	0	0	0	2	0	2
	2	0	97	18	4	2	121
	3	0	11	26	3	1	41
	4	0	0	0	6	3	9
	5	0	2	1	1	26	30
Total		0	110	45	16	32	203

### Histological reference standard

The PICTURE study employed TPM biopsy ± targeted biopsy of the suspected index lesion as previously described [4]. A TPM histological reference standard (as compared with prostatectomy reference) provides the opportunity to collect true-negative as well as true-positive cases. It has a reported sensitivity of 95% and negative predictive value of 95% for clinically significant cancers of volume > 0.5 cm^3^ and 76% sensitivity for all cancers [12–14].

An experienced (12 years) prostate pathologist analysed all biopsy cores blinded to the mp-MRI findings. Significant cancer was defined, based on previously reported TPM biopsy criteria [15], as the presence of any Gleason 4 tumour (primary or secondary pattern) or Gleason 3 + 3 tumour with a maximum cancer core length threshold 4 mm.

### Quantitative evaluation of mp-MRI

The test mp-MRI datasets were analysed with MIM Symphony Version 6.1 (MIM Software Inc.). Following radiologist scoring, the two radiologists were unblinded to each other’s mp-MRI reports and the histological reference standard. The radiologists, in consensus, manually contoured the regions of interest (ROI) on mp-MRI (T2-weighted images, apparent diffusion coefficient (ADC) maps and contrast early-enhanced T1-weighted images) to allow the extraction of quantitative mp-MRI metrics from regions of significant cancer and also from areas confirmed to have benign histology.

Specifically, where visible, the index lesion containing significant cancer within each zone (PZ and TZ) was contoured on each imaging sequence. Where no lesion was evident (even in retrospect) on mp-MRI and histological confirmed significant cancer was present, the radiologists contoured a 1-cm^2^ ROI at the histologically confirmed location of cancer. Where no lesion was visualised and/or no significant cancer present within a zone/lesion, the radiologists avoided the areas with histologically confirmed insignificant disease and contoured a 1-cm^2^ ROI at a histologically confirmed benign location.

The mean signal intensity (SI) of each ROI on the corresponding T2-weighted images, ADC maps and early T1 arterial contrast-enhanced images was recorded. T2 and early T1 arterial contrast-enhanced SI were normalised against a right obturator internus ROI to give normalised T2 (T2-nSI) and normalised early dynamic contrast-enhanced SI (DCE-nSI) parameters. The maximum enhancement (ME) parameter was also derived from the DCE signal enhancement time curve as previously reported [16].

### Application of LR diagnostic models

Previously derived [5, 6] zone-specific LR models (as below) were applied to calculate a predictive probability from the quantitative mp-MRI metrics extracted at each ROI anatomical location (ADC, T2nSI, ME for the TZ and, ADC, T2nSI and DCEnSI for the PZ).

$$ \mathrm{T}\mathrm{Z}\ \mathrm{model}:\ln \left(\mathrm{Odds}\right)=5.347+0.332\bullet \mathrm{ADC}-0.974\bullet \mathrm{T}2\mathrm{nSI}-1.730\bullet \mathrm{ME} $$

$$ \mathrm{PZ}\ \mathrm{model}:\ln \left(\mathrm{Odds}\right)=-2.441-0.968\bullet \mathrm{ADC}-0.200\bullet \mathrm{T}2\mathrm{nSI}+2.546\bullet \mathrm{DCEnSI} $$

Two separate probability thresholds were then used to classify an ROI anatomical location as positive or negative by the LR model.

The first threshold was based on the Youden index (*J*) applied to the published model derivation 1.5 T mp-MRI datasets [5, 6], where *J* is a function of sensitivity and specificity and is defined as the maximum vertical distance between the ROC curve and the diagonal line [17–20]. *J* occurs at the threshold probability for optimal diagnostic models classification ability. The maximum *J* within the PZ was *J* = 0.50, related to a probability threshold = 17%. For the TZ maximum, *J* was 0.56, with probability threshold = 31%.

The second threshold was set at a specificity of 50% from the ROC analysis performed previously on at the time of model derivation using the original 1.5 T mp-MRI dataset [5, 6]. At this specificity, the estimated probability threshold for the significant disease was 14% for the PZ and 23% for the TZ.

## Results

The ROC-AUC of the PZ and TZ LR models when applied to the test dataset mp-MRI ROI anatomical location quantitative metrics was 0.74 ± 0.04 (SD) and 0.68 + 0.04 (SD) respectively. The ROC-AUC for the zone-specific LR models was similar to that for the experienced radiologist’s Likert scores (Table 2). The agreement between radiologists A and B Likert scores, calculated using Cohen’s kappa, was good both in the PZ (*κ* = 0.625) and the TZ (*κ* = 0.609).

**Table 2:** ROC AUC of PZ and TZ LR models and comparative experienced radiologist performance for classification of significant cancer

			Lower bound	Upper bound
Peripheral zone				
PZ model	0.74	0.04	0.67	0.81
Radiologist A	0.78	0.03	0.72	0.84
Radiologist B	0.74	0.04	0.67	0.81
Transition zone				
TZ model	0.68	0.04	0.61	0.75
Radiologist A	0.69	0.04	0.61	0.77
Radiologist B	0.69	0.04	0.62	0.77

The sensitivities/specificities of the PZ model using the Youden index and 50% specificity probability thresholds were 0.84/0.57 and 0.90/0.51 respectively, and for the TZ model were 0.84/0.32 and 0.90/0.23 respectively (Table 3). Radiologists A and B had sensitivities/specificities of 0.91/0.40 and 0.90/0.33 in the PZ respectively and 0.58/0.75 and 0.60/0.65 in the TZ respectively (Likert ≥ 3 scored as positive).

**Table 3:** Performance of PZ and TZ models applied to 3 T mp-MRI PICTURE trial dataset at (i) Youdens index derived probability threshold and (ii) at set 50% specificity derived probability threshold

	Youdens index threshold		50% specificity threshold	
	Specificity	Sensitivity	Specificity	Sensitivity
PZ model	0.57	0.84	0.51	0.90
TZ model	0.32	0.84	0.23	0.90
Sub-analysis on equivocal group (Likert score 3)				
	Radiologist A	Radiologist B	Radiologist A	Radiologist B
	Specificity/sensitivity	Specificity/sensitivity	Specificity/sensitivity	Specificity/sensitivity
PZ model	0.42/0.75	0.52/0.61	0.39/0.90	0.48/0.78
TZ model	0.36/0.89	0.50/0.73	0.14/0.95	0.26/0.91

The PZ LR model with Youden index threshold classified 9% of patients as false positive and 20% as false negative; and with 50% specificity probability threshold, 5% as false positive and 23% as false negative. In comparison, using a Likert score threshold of ≥ 3 within the PZ as positive, radiologist A classified 5% of patients as false positive and 28% as false negative, whilst radiologist B classified 5% as false positive and 31% as false negative.

The TZ LR model with Youden index threshold classified 7% of patients as false positive and 38% as false negative and with 50% specificity probability threshold, 4% as false positive and 43% as false negative. In comparison, using a Likert score threshold of ≥ 3 within the TZ as positive, radiologist A classified 18% of patients as false positive and 14% as false negative, whilst radiologist B classified 18% as false positive and 20% as false negative.

**Fig. 4:**
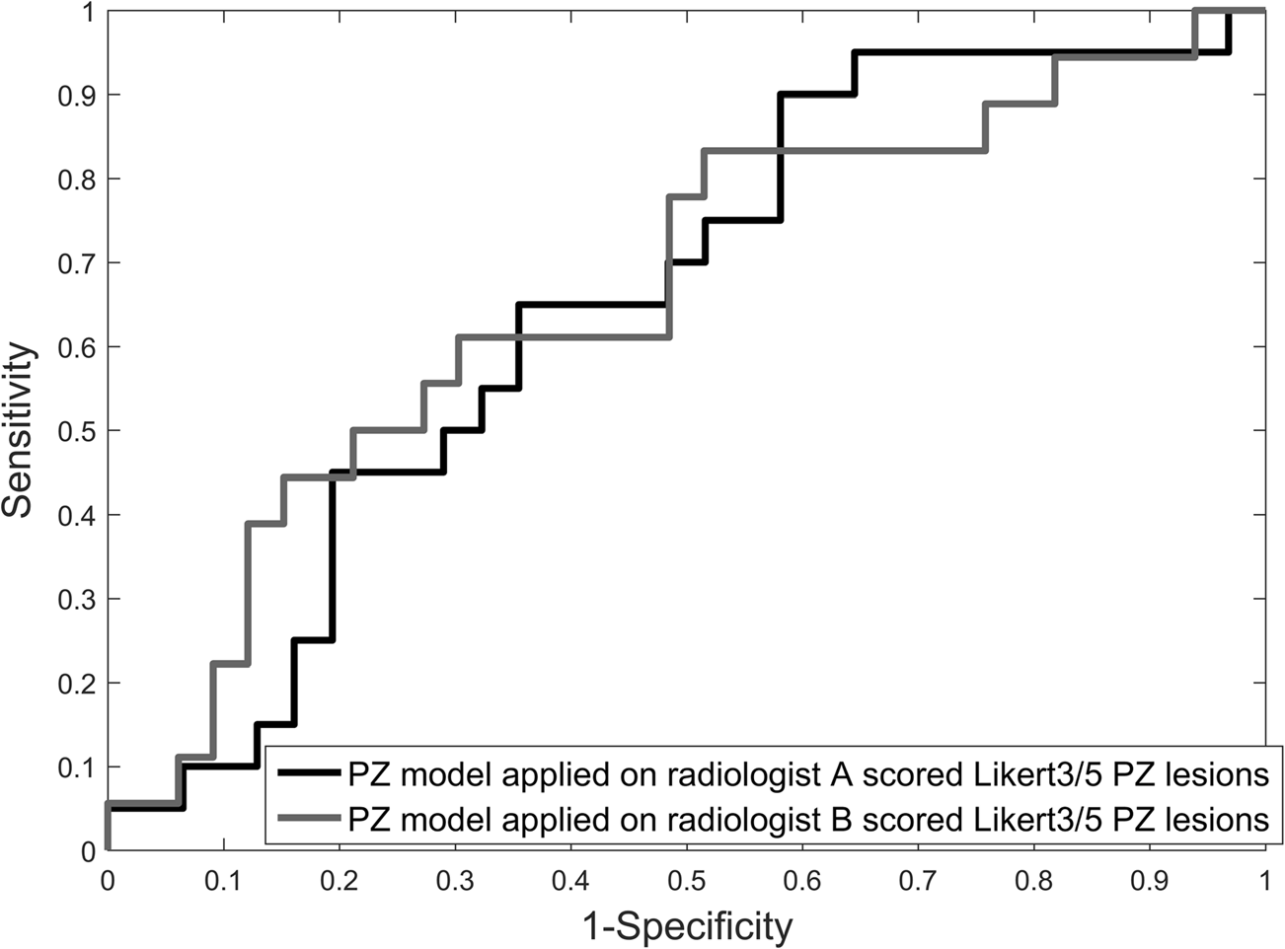
ROC curve for LR model classification of significant cancer for equivocal radiologist scored (Likert 3/5) PZ lesions from radiologist A, ROC-AUC = 0.65 (95% CI 0.50–0.80), and radiologist B, ROC-AUC = 0.67 (95% CI 0.51–0.82)

**Fig. 5:**
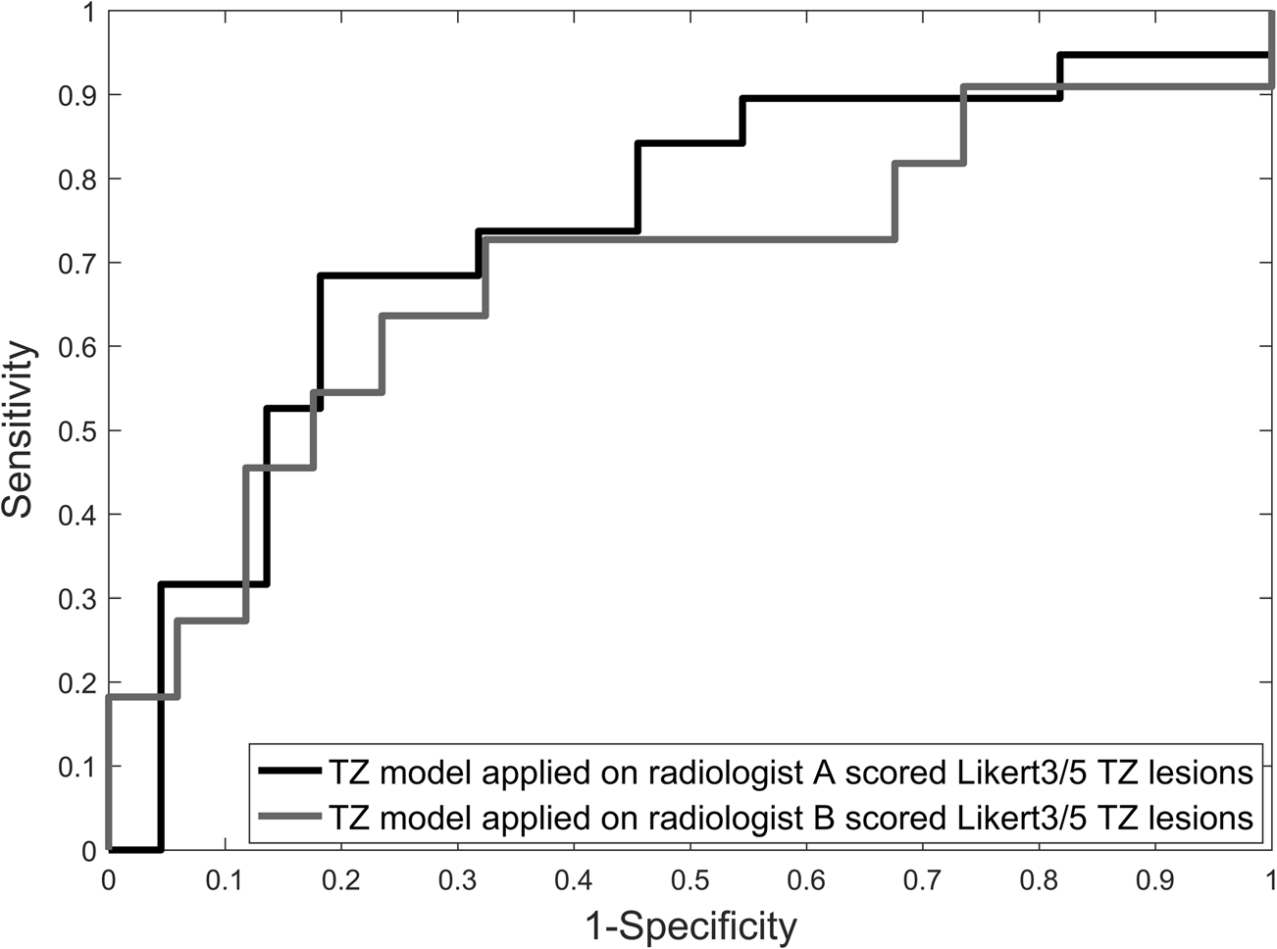
ROC curve for LR model classification of significant cancer for equivocal radiologist scored (Likert 3/5) TZ lesions from radiologist A, ROC-AUC = 0.74 (95% CI 0.58–0.90), and radiologist B, ROC-AUC = 0.69 (95% CI 0.62–0.77)

Radiologist A scored 51 PZ cases and 41 TZ cases as indeterminate (Likert 3/5). Application of LR models to the Likert 3/5 group yielded a ROC-AUC 0.65 (95% CI 0.50–0.80) for the PZ and ROC-AUC 0.74 (95% CI 0.58–0.90) for the TZ (Figs. 4 and 5). The model correctly classified 86% of PZ cases and 64% of TZ cases.

Radiologist B scored 51 PZ cases and 45 TZ cases as indeterminate (Likert 3/5). Application of LR models to the Likert 3/5 group yielded a ROC-AUC 0.67 (95% CI 0.51–0.82) for the PZ and a ROC-AUC 0.69 (95% CI 0.62–0.77) for the TZ (Figs. 4 and 5). The model correctly classified 92% of PZ cases and 62% of TZ cases.

For areas that were scored by the radiologists as (highly) likely or unlikely (Likert 1/5, 2/5, 4/5 and 5/5) to contain significant cancer, the zone-specific LR models had similar performance with the radiologists (Table 4)

- Radiologist A (Likert 1/5, 2/5, 4/5 and 5/5): The diagnostic model had an ROC-AUC = 0.77 (95% CI 0.68–0.85) in the PZ and 0.66 (95% CI 0.58–0.75) in the TZ. Radiologist A had an ROC-AUC = 0.80 (95% CI 0.72–0.87) in the PZ and 0.69 (95% CI 0.60–0.78) in the TZ.
- Radiologist B (Likert 1/5, 2/5, 4/5 and 5/5): The diagnostic model had an ROC-AUC = 0.73 (95% CI 0.65–0.82) in the PZ and 0.65 (95% CI 0.57–0.74) in the TZ. Radiologist B had an ROC-AUC = 0.74 (95% CI 0.65–0.83) in the PZ and 0.73 (95% CI 0.66–0.81) in the TZ.

**Table 4:** ROC AUC of PZ and TZ LR models and comparative experienced radiologist performance for areas that were scored by the radiologists as (highly) likely or unlikely (Likert 1/5, 2/5, 4/5 and 5/5) to contain significant cancer

	Area	Std. error	Asymptotic 95% confidence interval	
			Lower bound	Upper bound
PZ areas scored by radiologist A as Likert = 1/5, 2/5, 4/5 and 5/5				
PZ model	0.77	0.04	0.68	0.85
Radiologist A	0.80	0.03	0.72	0.87
PZ areas scored by radiologist B as Likert = 1/5, 2/5, 4/5 and 5/5				
PZ model	0.73	0.04	0.65	0.82
Radiologist B	0.74	0.04	0.65	0.83
TZ areas scored by Radiologist A as Likert = 1/5, 2/5, 4/5 and 5/5				
TZ model	0.66	0.05	0.58	0.75
Radiologist A	0.69	0.04	0.60	0.78
TZ areas scored by Radiologist B as Likert = 1/5, 2/5, 4/5 and 5/5				
TZ model	0.65	0.05	0.57	0.74
Radiologist B	0.73	0.04	0.66	0.81

## Discussion

There were four principal observations. Firstly, we confirm that the performance of both PZ and TZ LR models for classification of patients with significant tumour was comparable to experienced radiologists.

Secondly, we highlight that zone-specific mp-MRI LR models may help classify equivocal radiologist-scored areas (Likert 3/5).

Thirdly, the examined zone-specific mp-MRI LR models reliably classified significant cancers both in the PZ and the TZ (the percentage of patients classified as false positives ranged from 2 to 10%). In agreement with the PI-RADS v2 guidelines [21], the contribution of ADC is higher than the contribution of T2 in the PZ model, whereas for the TZ model, T2 has higher contribution than ADC.

Fourthly, by applying 1.5 T mp-MRI-derived LR models to an independent 3 T mp-MRI dataset, we have confirmed the external validity and generalizability of the previously published models [5, 6]. The generalizability can be attributed to the selected quantitative MR parameters, which are calculated as relative signal changes hence are less affected by the differences during data acquisition between the training (1.5 T magnet) and the independent (3 T magnet) cohort. This finding is also supported by a previous study [6] where the examined TZ diagnostic model was applied on a temporal cohort of TZ prostate cancer patients imaged at the same 1.5 T scanner. This study showed similar results of the temporal validation (ROC-AUC = 0.67) with the independent validation illustrated here (ROC-AUC = 0.68).

Our study evaluated previously reported 1.5 T mp-MRI zone-specific diagnostic model performance on an independent cohort of patients scanned on a 3-T MR system to evaluate their ability to aid radiologists. Separate validation of the models was performed for areas identified as equivocal that may practically aid experienced radiologists in predicting tumour. Previous work from our group [22] also reported the reverse scenario where zone-specific LR models were trained in the 3 T mp-MRI dataset and applied on the 1.5 T mp-MRI dataset. The reported ROC-AUCs were 0.76 (%CI 0.68–0.97) in the PZ and 0.77 (%CI 0.66–0.89) in the TZ. Following a sub-analysis, for the cases that were scored as indeterminate, the ROC-AUCs were 0.72 (%CI 0.50–0.94) in the PZ and 0.77 (%CI 0.55–0.96) in the TZ. The reported ROC-AUCs are similar to the ones reported in this work especially in the PZ, but even in the TZ, the ROC-AUC differences are not significantly different (*p* > 0.05).

In the current study, even though expert radiologists reported the mp-MRI, diagnostic accuracy remained modest overall, and almost a quarter of regions evaluated were classified as equivocal for clinically significant cancer. This is in accordance with other studies where up to 40% of the cases were reported as indeterminate (using either the PI-RADS or the Likert scoring) for the presence of cancer by the radiologist after reviewing mp-MRI dataset [23]. Approximately 40% of the cases classified as equivocal, contained significant cancer on TPM histology in the PZ, and 46% of these contained significant cancer on TPM histology in the TZ.

We found similar ROC-AUCs for the radiologist consensus score and the derived mp-MRI diagnostic models. At first glance, this suggests that the models would not benefit experienced radiologists working in consensus. However, the ROC-AUC for radiologists’ evaluation does not reflect the uncertainty of equivocal scores. When the diagnostic models were evaluated on the radiologist A equivocal ROIs (51 in the PZ and 41 in the TZ), 86% of PZ model predicted cases and 64% of TZ model predicted cases were correctly identified. Similarly, when the diagnostic models were evaluated on the radiologist B equivocal ROIs, 92% of PZ model predicted cases and 62% of TZ model predicted cases were correctly identified. Following a sub-analysis on the cases scored as indeterminate by the radiologists the percentages of false positives predicted by the diagnostic models ranged from 2 to 10%. These results indicate that the zone-specific diagnostic models are trustworthy when classifying an indeterminate case as significant cancer and that the diagnostic model may indeed have the potential to aid even experienced radiologists.

Evidence is mounting for the use of mp-MRI to triage patients prior to performing a prostate biopsy. Negative mp-MRI is associated with about 1–2 in 20 risks of clinically significant disease [24], whereas positive mp-MRI is associated with about 3 in 10 risks of clinically insignificant disease [25, 26]. Equivocal mp-MRI scores would tend to be biopsied. A tool to assist radiologists to avoid an equivocal category and correctly identify those patients with a low risk of clinically significant tumour would be of considerable value. Using the clinically relevant probability threshold on the indeterminate cases, the examined zone-specific mp-MRI models had (i) sensitivity 90% in the PZ and 95% in the TZ for radiologist A and (ii) sensitivity 78% in the PZ and 91% in the TZ for radiologist B.

Our study has several limitations. We were reliant upon visual matching of the Barzell zone histology on TPM and ROIs on mp-MRI. Therefore, the results may be influenced by misregistration errors. Although no biopsy is free from sampling error [27], we used TPM to address as much of the systematic error inherent to TRUS biopsy as possible [13, 28]. TPM also enables sampling in the anterior gland. Radical prostatectomy specimens could not act as an alternative for this study, as they would suffer from a large amount of selection bias given that they exclude men with no cancer and represent a minority of all men diagnosed [29]. Moreover, they also present technical challenges of accounting for distortion and gland shrinkage during fixing and the tissue loss in preparing the whole mount which is not trivial [30]. The relative merits of each reference standard have been considered before, but we believe TPM to be preferable in this patient cohort as it includes patients negative for cancer but presenting an elevated PSA.

## Conclusions

In summary, we have assessed the performance of zone-specific mp-MRI diagnostic models for classification of peripheral and transition zone signal, demonstrated their similar performance to an experienced radiologist consensus and highlighted their ability to classify equivocal areas.

## Abbreviations

AUC: Area under the curve
CAD: Computer-assisted diagnosis
DCE: Dynamic contrast-enhanced
DCE-nSI: Early contrast-enhanced T1 signal intensity
DWI: Diffusion-weighted imaging
LR: Logistic regression
ME: Maximum enhancement
mp: Multi-parametric
PSA: Prostate-specific antigen
PZ: Peripheral zone
ROC: Receiver operator characteristic
ROI: Region of interest
SI: Signal intensity
T2-nSI: Normalised T2 signal intensity
TPM: Template mapping biopsy
TRUS: Transrectal ultrasound-guided biopsy
TZ: Transition zone
